# Biophysical modelling as a tool for advancing plant science and engineering

**DOI:** 10.3389/fpls.2025.1693857

**Published:** 2025-12-04

**Authors:** Debanik Deb, Bandan Chakrabortty

**Affiliations:** Theoretical and Computational Biology Lab, School of Biology, Indian Institute of Science Education and Research (IISER) Thiruvananthapuram, Thiruvananthapuram, Kerala, India

**Keywords:** plant developmental biology, biophysical modelling, computational plant biology, plant mechanobiology, plant systems biology, synthetic biology, plant engineering

## Abstract

Plant growth, development, and physiology are governed by complex processes operating across scales spanning from molecular, cellular, tissue, and whole organism. Experimental studies have uncovered key mechanisms underlying hormone signalling, gene regulation, metabolism, and environmental responses. However, integrating this information into a unified framework to understand the mechanistic origin, evolution, and robustness of plant form and function remains challenging. Mathematical and computational modelling provides powerful tools to address this challenge, where simulation and predictions offer strategic guidance for efficient experimental search of missing information underlying plant processes. In this review, we discuss a broad spectrum of modelling approaches, including reaction–kinetic and Boolean network models for molecular and genetic regulation; mechanical and geometry-based models for tissue growth and morphogenesis; metabolic and constraint-based models for resource allocation; and hydraulic and electrophysiological models for physiological transport processes. We also propose that by combining different modelling strategies, researchers can develop predictive tools for biotechnological applications, including enhancement of stress tolerance, efficient use of nutrients, regenerative tissue engineering, and biomass productivity.

## Introduction

Plants are dynamic living entities with remarkable complexity in their fundamental biological processes, including growth, development, and physiology. These processes occur across multiple scales, ranging from molecular interactions within cells to the emergence of macroscopic patterns of organs (Iwamoto and Asaoka, 2024). Understanding these intricacies is necessary not only for advancing our knowledge of fundamental plant biological processes but also for addressing global issues such as sustainable agriculture, food security, and climate change (Jeger et al., 2021). Despite advances in experimental techniques, capturing the highly dynamic and multiscale nature of plant development remains challenging. Experimental studies often focus on individual aspects of plant development, like the specific role of hormones in regulating growth(Zhang et al., 2022), the genetic control of cell differentiation, and epigenetic regulations (Takatsuka and Umeda, 2015), or the impact of environmental stress on crop yield (Ramegowda et al., 2024). However, understanding how these pieces of information are coordinated in a controlled fashion that guides coordinated growth and development is challenging. Over the past few decades, mathematical and computational modelling have emerged as a powerful tool for addressing this key challenge by allowing integration of experimentally accessible information into a theoretical framework, which not only provides mechanistic insights into complex multifaceted aspects of plant growth and development but also offers predictions for efficient experimental search. In the current era of rapid climate change, plants face increasingly unpredictable environmental conditions, and such a combined approach will fast-track designing resilient crops and sustainable agriculture strategies (Timlin et al., 2024). One of the key strengths of computational modelling lies in its ability to reveal fundamental organizing principles that can be leveraged to connect processes happening at different scales, e.g., a tissue-scale process can be explained by interactions happening at cellular scales. Translating these integrative ideas into implementation has led researchers to adapt a wide range of mathematical and computational frameworks, of which many have originated outside biology, to explore the mechanisms of plant growth and development.

In a seminal work, Turing proposed a mathematical concept that provides insight into the chemical basis of morphogenesis, where various biochemical compounds were defined as morphogens (Turing, 1952). This study provided a framework for biological problems through ordinary differential equations (ODEs), which are used to explain the formation of hormone patterning required for the maintenance of meristematic cell identities. The process of growth and deformation involves the deformation of individual cells. To study the mechanistic origin of cell deformation, attempts have been made to use the energy minimization-based vertex model, a model originally developed to study bubble packing in foams (Weaire and Rivier, 1984). For tissue-scale processes, individual cell identities become irrelevant, and tissue can be approximated by a continuous membrane. Under such an assumption, many tissue-scale processes have been studied using a Finite Element model, a model initially designed for structural engineering, using matrices for stiffness analysis (Clough, 1960). Optimal cell growth and deformation involve efficient use of nutrients and energy, thus requiring strict constraints on the use of available resources. Considering this, the necessary metabolic flux for nutrient redistribution has been predicted through the Constraint-Based Model, whose foundation lies in linear programming, i.e., optimizing an objective function subject to a set of linear inequalities (Dantzig, 1947). While adaptive metabolic flux allows efficient use of nutrients, this requires the maintenance of certain nutrient levels via active transport of nutrients/ions between neighbouring cells. Observing a close resemblance to the ion transport mechanism in neurons, effort is being made to understand the ion flux dynamics using the groundbreaking Hodgkin-Huxley model, which describes how action potentials in neurons are initiated and propagated (Hodgkin and Huxley, 1952). These studies indicate that seeking deeper insight into plant growth, development, and physiology requires the use of classical mathematical or computational concepts, which seem to have little or no relevance in the early days of understanding biological systems or processes. Notably, such cross-disciplinary approaches toward biological understanding have started to gain importance with the increasing availability of experimental data, primarily due to technological advances in microscopy and storage facilities. Accordingly, in the last few decades, the interdisciplinary approach has taken a big leap with the explicit development of state-of-the-art mathematical/computational frameworks specifically to study plant biology. While most of these modelling frameworks help us to identify key fundamental concepts underlying the complexity of plant processes, they also have their own limitations. For example, many of the existing modelling approaches, under specific assumptions, can explain a plant process, but fail to predict an outcome under changes in parameter space. Such models lack thorough sensitivity analyses for key parameters, and no/incomplete validation against experimental data constrains their reliability. Another important aspect is the lack of scalability and integration of the various modelling frameworks. For example, a model that can explain a tissue-scale phenomenon depends on cellular input, which can be explained by a model specific to an associated cellular or molecular process (Gao and Zhao, 2024). However, capturing the true multiscale nature of plant growth and development requires a unifying framework that seamlessly integrates processes occurring at whole-organism scales, i.e., the molecular, cellular, and tissue scales, with the inclusion of environmental response. Furthermore, the modelling framework needs to be quantitative, allowing integration of experimental data, both to advance our basic knowledge and apply it in plant engineering.

In this review, we aim to visit the widely used computational and mathematical modelling techniques to study plant growth, development, and physiology that have significantly advanced our understanding of plant biology. The review is sketched by discussing modelling frameworks developed along the line of the progression of plant growth and development from the establishment of the early meristem, a collection of undifferentiated cells required for tissue specification. We begin by discussing reaction-kinetic and Boolean network models that capture gene regulation and signalling in the root and shoot meristem. Subsequently, we cover mechanical and geometry-based models, which explain, post division and differentiation of meristematic cells, how coordinated cell growth leads to the emergence of nontrivial plant organ morphology. Next, we examine metabolic and constraint-based models that address strategies for efficient resource allocation required to maintain growth homeostasis and adapt to stress, such as nutrient scarcity, oxidative damage, or environmental change. Following this, we examine hydraulic and electrophysiological models that elucidate how plants maintain the sustained transport of metabolic resources, essential for metabolism, survival, and adaptation to environmental fluctuations. Finally, we propose integrative frameworks for biotechnological applications, including the incorporation of AI-based approaches that promise to enhance the predictive power of modelling frameworks.

## Meristem and stem cell regulation

**Figure 1:**
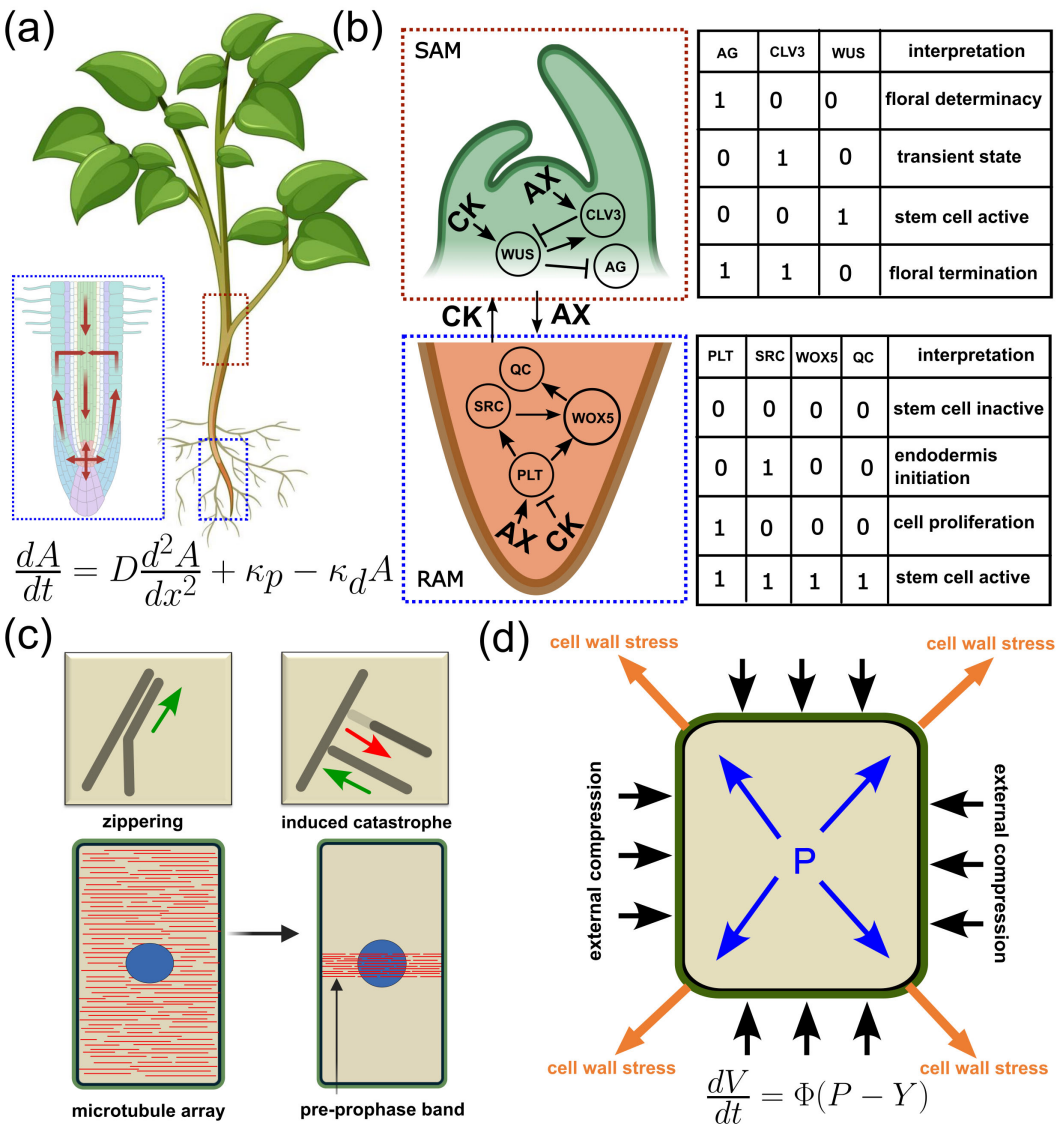
Models for regulatory networks and force-generating processes. **(a)** Kinetic model of auxin transport and reflux loop in the plant Root Apical Meristem (RAM), highlighted by the blue dashed box. Flow direction is shown by red arrows, where auxin dynamics follow a kinetic equation described by concentration (A), synthesis rate (k_p_), degradation rate (k_d_), and diffusion constant (D). **(b)** Boolean Model describing gene regulatory interactions in the Shoot Apical Meristem (SAM), highlighted by the red dashed box. Here, WUSCHEL (WUS) expression at time t+1 is regulated by CLAVATA3 (CLV3) and AGAMOUS (AG) as shown in the truth table and described by the Boolean operation, WUS = NOT CLV3 AND NOT AG. In RAM, highlighted by the blue dashed box, WOX5 expression is controlled by SCARECROW (SCR) and QUISCENT CENTER (QC) as shown in the truth table and described by the Boolean operation, WOX5 = SCR AND NOT QC. **(c)** A model for stochastic simulation of plant cortical microtubules (MT), where MT-MT interactions (e.g., zippering, induced catastrophe) can be simulated via an event-driven algorithm to understand the formation of aspontaneous MT-array (shown in red). The MT-array initially surrounds the nucleus (shown in blue), and later transitions into a distinct pre-prophase band that marks the site of cell division. **(d)** Lockhart Model for the regulation of cell volume (V) expansion during plant cell growth, illustrating the interaction of turgor pressure (P, shown by blue arrows), wall extensibility (Φ), and yield threshold (Y), which represents the minimum turgor pressure required for a plant cell to undergo irreversible expansion. Force balance is reached through external counteractive forces (shown in black arrow) generated by wall stress (shown in orange arrow).

We start by asking how plants maintain meristem, a distinct population of stem cells, necessary for sustained growth and regeneration. To gain insight into this process, in this section, we are going to discuss models that help us understand one of the most fundamental processes, i.e., stem cell regulation in plants. Starting from a single embryonic cell, in the absence of significant growth, oriented division cells play a decisive role in the early-stage morphology of the plants. Following the initial few rounds of division, plant cells gradually gain root-specific or shoot-specific identities mediated by two distinct stem cell clusters known as the Root Apical Meristem (RAM) and the Shoot Apical Meristem (SAM). Root and shoot are distinct both morphologically and functionally, and to preserve this, the maintenance of the respective meristematic state is crucial. Here, the plant hormone auxin plays a pivotal role in maintaining the biochemical network that preserves the respective meristematic cell identities. An excellent way to describe the dynamics of auxin concentration is through Kinetic Models (KM, **Table 1**- Eqn. 1), which incorporate ODE-based reaction kinetics (**Figure 1a**). In the KM equation for auxin transport, the diffusion coefficient (D) determines passive auxin spread, the production rate ( $K_{p})\;$ quantifies the auxin biosynthesis and degradation rate ( $K_{d})\;$ corresponds to the activity of auxin-degrading enzymes. Using KM, it has been shown that the interplay between auxin transport and its impact on cell division and expansion contributes to RAM maintenance (Grieneisen et al., 2007). In this context, a recent study presents a model that integrates the thermodynamic properties of the membrane-associated auxin transporter to describe the establishment and regulation of transmembrane auxin gradients (Geisler and Dreyer, 2024). However, in KMs, quantitative analyses show that increasing the diffusion coefficient leads to wider and flatter auxin gradients, while a high degradation rate restricts the auxin domain, directly impacting the predicted size of the meristematic region. These predictions indicate that even modest changes in these parameters can alter stem cell domain boundaries and the overall robustness of meristem maintenance. In addition, these models failed to capture fluctuations in auxin level, which is known to have important developmental consequences. To overcome such limitations, a complementary Particle Based Modelling (PBM) approach has been developed, where auxin molecules are treated as distinct entities with random fluctuations in their dynamics. This approach offers long-distance auxin transport and a more dynamic view of auxin-mediated controls in plant growth (Renton et al., 2012). These approaches reveal that increasing the noise in particle movement can lead to developmental plasticity, as observed during environmental adaptation or stress.

**Table 1:** Model equations and software tools.

Model name	Model equation	Model parameters	Software
Kinetic	$\frac{dc}{dt}=D\frac{d^{2}c}{dx^{2}}+\kappa_{1}-\kappa_{2}c$ (1)	c : concentration species D : diffusion coefficient $\kappa_{1\;\;\;\;\;\;}$ : production rate $\kappa_{2\;\;\;\;\;\;}$ : degradation rate	COPASI, Tellurium, PySB, OnGuard, VCell
Enzyme Kinetic	$v=\frac{V_{max}S}{K_{m}+S}$ (2)	v : reaction velocity $V_{max}$ : maximum reaction velocity S : substrate concentration $K_{m}\;\;\;$ : Michaelis constant	
Boolean Network	$x_{i}(t+1)=f_{i}(x_{j1}(t),x_{j2}(t),\;\ldots )$ (3)	$x_{i}(t)$ : state of gene i at time t j1, j2 : regulators that control i $f_{i}$ : logical rule	BoolNet, GINsim, PyBoolNet
Lockhart	$\frac{dV}{dt}=\phi (P-Y)\;$ (4)	V : cell volume ϕ : cell wall extensibility P : turgor pressure Y : yield threshold	Custom ODE solvers
Vertex	$E=\sum_{i}\beta {\left(A_{i}-A_{0}\right)}^{2}+\sum_{i}\alpha l_{i}$ (5)	E : system energy β : area elasticity modulus $A_{i\;\;\;\;}$ : actual cell area $A_{0\;\;\;\;}$ : target cell area α : line tension $l_{i}\;\;\;\;\;$ : length of cell edge	Tyssue, VirtualLeaf, Tissue Analyzer
Finite Element	Ku=F (6)	K : stiffness matrix u : displacement vector of nodes F : force/load vector	Abaqus, COMSOL, FEniCS
Vertex Element	$\sigma +\tau \frac{d\sigma }{dt}=E\epsilon +\eta \frac{d\epsilon }{dt}$ (7)	σ : stress τ : stress relaxation time E : elastic modulus/stiffness ϵ : strain η : viscosity/dashpot coefficient	Julia DifferentialEquations.jl
Mass Spring	$\frac{d^{2}x_{i}}{dt^{2}}={F_{i}}^{spr}-\gamma \frac{dx_{i}}{dt}+{F_{i}}^{ext}$ (8)	$x_{i\;\;\;\;\;\;\;\;}$ : position of node ${F_{i}}^{spr}$ : net spring force γ : viscous drag ${F_{i}}^{ext}$ : external forces	Blender physics, MorphoMechanX
Constraint-Based	$max:\;Z=c^{T}v$ S.v=0 ${v_{i}}^{min}\leq v_{i}\leq {v_{i}}^{max}$ (9)	Z : objective function c : individual contribution to Z v : flux vector S : stoichiometric matrix ${v_{i}}^{min},{v_{i}}^{max}$ : flux bounds	COBRA Toolbox, Cameo
Proteome Constrained	$v_{j}\leq k_{cat,j}\leq E_{j}$ (10)	$v_{j}$ : reaction flux $k_{cat,j}$ : catalytic turnover number $E_{j}$ : enzyme concentration	GECKO, AutoPACMEN
Hagen-Poiseuille	$Q=\frac{\pi \text{Δ}PR^{4}}{8\eta L}$ (11)	Q : volumetric flow rate ΔP : pressure between tube ends R : tube radius η : dynamic viscosity of the fluid L : tube length	TREES, SurEau
Hydraulic-Electrical	$Q=\frac{\text{Δ}\psi }{R}$ $C\frac{d\psi }{dt}=Q_{in}-Q_{out}$ (12)	Q : volumetric flow rate Δψ : difference in water potential R : hydraulic resistance C : hydraulic capacitance $Q_{in}\;\;$ : inflow rate of water $Q_{out}$ : outflow rate of water	NEURON
Nutrient Uptake	$J_{mass}=\rho v\;$ $J_{diff}=-D\frac{d\rho }{dx}$ $J_{total}=J_{mass}+J_{diff}$ (13)	$J_{mass}\;\;\;\;$ : mass flow flux ρ : nutrient concentration v : nutrient transport velocity $J_{diff\;\;\;\;\;\;\;}$ : diffusion flux D : diffusion coefficient $J_{total\;\;\;\;\;}$ : total flux	PlantHydraulics.jl
Hodgkin-Huxley	$C_{m}\frac{dV_{m}}{dt}=-g_{L}(V_{m}-E_{L})$ (14)	$C_{m\;\;\;\;\;}$ : membrane capacitance $V_{m}\;\;\;\;$ : membrane potential $g_{L\;\;\;\;}\;$ : leak conductance $E_{L}\;\;\;$ : reversal potential for the leak	NEURON

List of commonly used mathematical forms (i.e., equations) and software tools for studying plant growth, development, and physiology.

In the context of SAM, using a quantitative and dynamic mathematical model, it has been shown how the SAM maintains homeostasis under varying environmental and developmental conditions (Geier et al., 2008). The underlying gene regulatory networks have been described by Boolean Network Models (BNMs, **Table 1**- Eqn. 3), where the expression of genes or proteins ( $x_{i}\;)\;$ are represented as nodes with binary states (0 or 1), which are determined by local rules (AND, OR, etc.) that govern gene activation ( $x_{i}=1$) or repression ( $x_{i}=0$). This study integrates experimental data on meristem domain sizes and cell division rates to construct a set of coupled ODEs representing the feedback loop between WUSCHEL (WUS) and CLAVATA3 (CLV3). The model accurately predicts known phenotypes such as CLV3 and WUS overexpression, and predicts that under steady environmental conditions, the stem cell pool size reaches a stable equilibrium (**Figure 1b**). Implementing a similar computational approach, it has been shown that maintenance of the WUS gradient is essential for the regulation of stem cell number (Yadav et al., 2011). The BNM associated with the WUS-CLV3 feedback in SAM employs logical rule sets and activation thresholds to mimic genetic switch behaviour. Systematic variation of binary rules or threshold values helps interpret how robust gene circuits underpin meristem function and how mutations or perturbations may destabilize stem cell pools. Building upon this feedback loop, a more comprehensive mathematical modelling framework has been introduced, which offers a multi-dimensional view of SAM homeostasis, showing how local and global cues are integrated to precisely pattern and stabilize the stem cell niche (Liu et al., 2020). This study underscores the role of synergistic signalling and paradoxical feedback in enhancing the robustness and flexibility of developmental systems.

Meristem cells are not terminally differentiated and can adopt multiple fates, i.e., they have the potential to become a tissue-specific cell type through differentiation, e.g., stem cells at the root meristem can differentiate into root endodermal cells or root epidermal cells, and so on. Such a cellular decision-making process involves interactions of specific genes, often forming regulatory networks (Karanam and Rappel, 2022). Using BNM, gene expression data have been integrated into a dynamic gene regulatory network to explore the robust cell fate identity of root stem cell niches (Velderraín et al., 2017). In summary, the models discussed in this section show how hormone signalling and genetic networks ensure stable stem cell niches, providing the foundation for subsequent tissue growth.

## Growth and patterning

Once meristems are established, the next question is how plants coordinate growth and patterning across tissues. In this section, we are going to visit models that explain the oriented cell division and subsequent growth leading to the emergence of nontrivial plant morphology. Following the establishment of RAM and SAM, plants achieve a remarkable ability to instruct cell division and subsequent differentiation of the stem cells to add new cells of a specific tissue type. While cell differentiation in plants can be explained by BNMs, to understand the molecular basis of cell division, we need to understand the mechanism that drives the formation of the cell cortex-specific microtubule array, as the orientation of this array is a good predictor of cell division orientation. Notably, cell geometry directly affects the microtubule dynamics and, by extension, array orientation. Here, to simulate the complex coupling between microtubule dynamics and cell geometry, a novel computational framework has been developed that allows the simulation of microtubule dynamics on experimentally extracted plant cell surfaces (Chakrabortty et al., 2018a). This framework allows the implementation of basic microtubule interaction rules and the study of collective microtubule dynamics on realistic place cell surfaces (**Figure 1c**). Using this modelling framework, it has been shown that cell division orientation for nongrowing cells is governed by a complex interplay between cell geometry and self-organizing principles that control microtubule dynamics (Chakrabortty et al., 2018b). Post division and differentiation to a specific tissue type, the daughter cells show significant growth, which is driven by hydrostatic pressure from cytoplasmic fluid (i.e., turgor pressure). Notably, cell volume expansion is limited by the mechanical properties of the cell wall and its capacity to yield under mechanical stress. This implies that cell volume expansion depends on how much the wall can stretch, i.e., the ability of the cell wall to yield under turgor pressure sets the limit for volume expansion. A quantitative framework for studying turgor pressure-driven irreversible cell expansion is the Lockhart Model (**Table 1**- Eqn. 4), in which the cell wall is assumed to act as a linear viscoelastic material (Lockhart, 1965). The associated model equation links cell growth rate, expressed as changes in volume ( V), to wall extensibility ( ϕ), which is governed by cell-wall loosening enzymes and microfibril organization that can be experimentally manipulated through genetic or pharmacological means. Thus, measuring V while modulating ϕ through altered cellulose microfibril orientation provides a direct route to test and refine models of cell expansion and morphogenesis. Turgor-driven cell growth can be influenced by changes in the mechanical properties of the cell wall via microtubule array-guided deposition of cellulose microfibrils that steer anisotropic cell growth (**Figure 1d**). In this model, wall extensibility is a critical parameter, as variations in ϕ, influenced by cellulose microfibril organization, can strongly impact predicted growth rates. Taking this into account, recent models incorporate the mechano-sensitive angle of cellulose microfibril deposition, which interacts with the turgor pressure and matrix stiffening to influence the cell growth pattern (Chakraborty et al., 2021). Using this model, it has been shown how plant roots respond to mechanical obstacles during the early growth stages(Quiros et al., 2022). Notably, plant cells cannot migrate, as they are cemented together through the shared rigid cell wall, thus forming plant tissue. This strong mechanical connection allows individual cell growth to be coupled with the neighbouring cells, therefore requiring growth coordination among the cells for the emergence of robust plant morphology. However, the Lockhart model captures growth at the single-cell level; therefore, it lacks multicellular growth coordination, where local wall extensibility and turgor pressure gradients collectively can shape tissue morphology.

**Figure 2:**
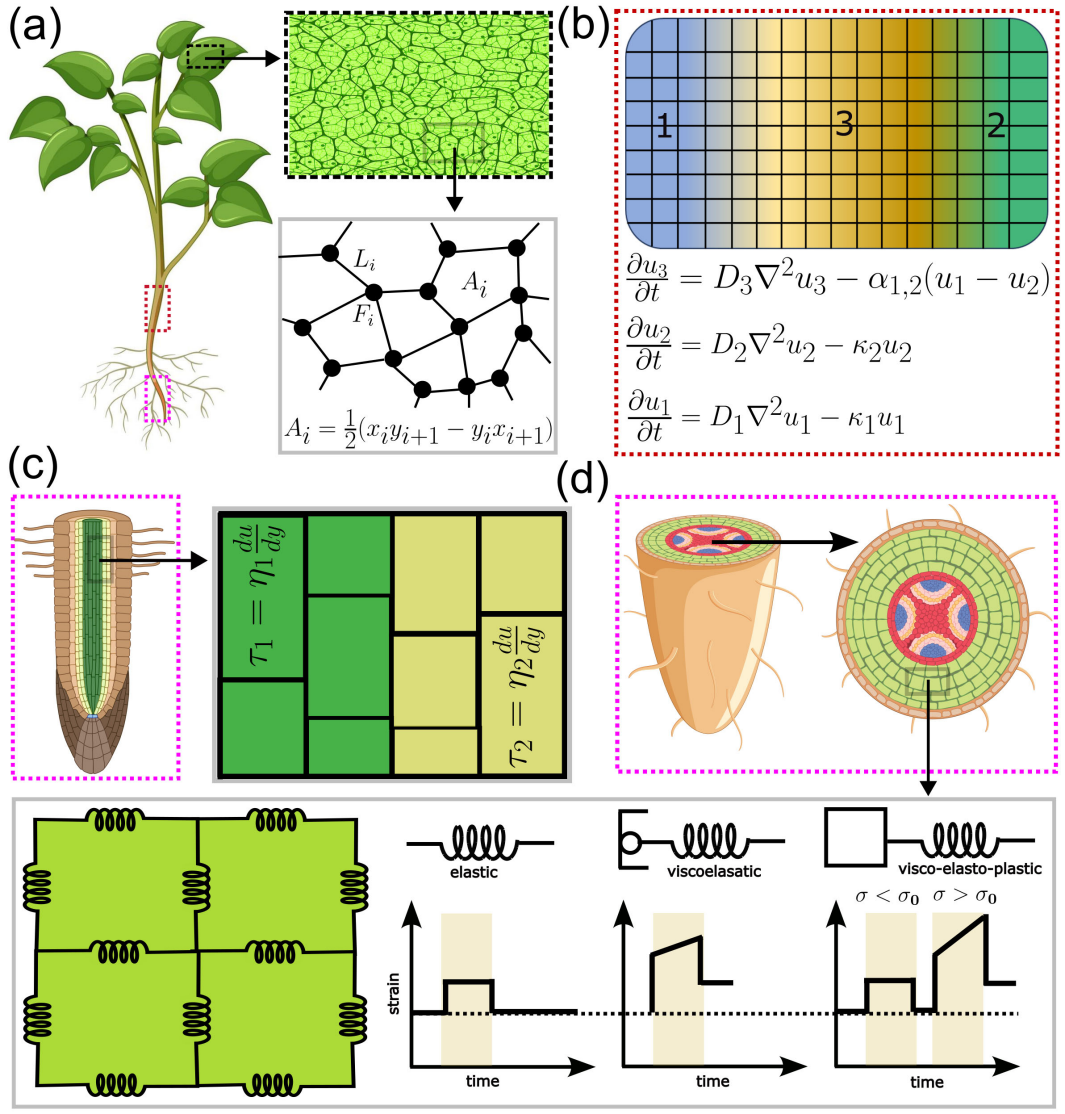
Models for integrating cell mechanics and material properties. **(a)** Vertex model for studying the mechanical response of plant leaf tissue, highlighted by the black dashed box. Cells are described as polygons of vertices (black dots) and force balance at each vertex (F_i_) is reached via vertex movement, resulting in cell deformation, i.e., changes in cell area (A_i_) or edge length (L_i_), which depend on the vertex positions (x_i_, y_i_). **(b)** Finite element model for diffusion of biochemical species (u_i_) across different zones of plant shoot tissue, highlighted by the red dashed box in panel (a). Concentration of biochemical species (u_i_) possesses zone-specific (i = 1,2,3) dynamics governed by associated diffusion (D_i_) and decay (K_i_) coefficients. Dynamics in one zone can be coupled via the coupling term a_i,j_(u_i_ − u_j_). **(c)** Vertex element model for studying the mechanical response of multi-layered plant root tissue, highlighted by the magenta dashed box in panel (a). In addition to the polygonal representation of cells, this model accounts for tissues with different stiffness, whose impact on tissue deformation (i.e., strain rate u or its spatial gradient du/dy) is modelled through layer specific (i = 1,2) shear stress (τi) and dynamic viscosity (ηi). **(d)** Mass-Spring model for studying biomechanical interactions among cells in the plant root tissue, highlighted by the magenta dashed box in panel (a). In its cross-sectional representation, the junction of cells with a common cell wall is described by springs that respond elastically or viscoelastically to a low value of mechanical stress (σ < σ_0_, yield stress). For σ > σ_0_, the response becomes visco-elasto-plastic, leading to irreversible strain/deformation.

A modelling framework that enables investigation of coordinated growth by accounting both cell geometry and cell-cell mechanical interactions is the energy minimization principle-based Vertex Model (VM, **Table 1**- Eqn. 5), in which the plant tissue is represented as a network of interconnected polygons of vertices, representing cells that dynamically change their shape under the influence of mechanical force (**Figure 2a**). In this model, the energy ( E) of the system is expressed in terms of changes in cell area ( $A_{i}$) or changes in cell edge length ( $L_{i}$), which are functions of the vertex position ( $x_{i}$, $y_{i}$). The state of minimum energy, i.e., mechanical equilibrium, is achieved by force balance at each vertex, which is reached via changes in vertex position, resulting in cell deformation. This framework has been used to investigate stress-dependent cell division in plant tissue, revealing how mechanical feedback generates reproducible division patterns necessary for robust plant morphology (Louveaux et al., 2016). In the context of understanding the mechanics behind plant morphology, consideration of individual cell mechanics involves many biophysical parameters that are not accessible. In VMs, parameters like cell wall stiffness, turgor pressure, and adhesion strength determine how plant cells deform, divide, and coordinate growth. Experimentally, these parameters can be probed via AFM, osmotic assays, or adhesion markers, but *in vivo* values vary across tissues and time. A small change in these parameters can drastically alter predicted division patterns or tissue geometry, highlighting the gap between measurable variability and model precision. Moreover, dynamic wall remodelling and mechanical anisotropy are difficult to capture explicitly. Hence, despite their utility in linking mechanics to morphogenesis, vertex models remain constrained by parameter uncertainty and limited experimental validation. This limitation can be overcome by the Finite Element Model (FEM, **Table 1**- Eqn. 6), where tissue is divided into small, interconnected elements (e.g, triangles described by three nodes), thus simplifying the study of biomechanics that focuses on local regions (**Figure 2b**). FEM can be employed to perform ODE-based simulation of the spatiotemporal diffusion of biochemical species within plant tissues, where concentration fields evolve according to zone-specific diffusion and decay parameters. This allows capturing biochemical-mechanical interactions underlying developmental patterning and signalling dynamics. FEM has also been widely applied to study the mechanical aspects of plant growth and morphogenesis. In this approach, the displacement ( u) of the nodes is computed by solving a set of equations described in a matrix form Ku=F, where K is the stiffness matrix (consisting of parameters like Young’s modulus, Poisson’s ratio) and F is the force/load vector. Many of the parameters in FEM correspond to measurable mechanical traits such as elasticity differences among epidermal and cortical layers that can now be quantified using modern indentation and live-imaging techniques, offering opportunities for model validation. An interesting application of FEM can be observed in understanding how the mechanical feedback helps control the growth for the appropriate shaping of organs, e.g., the sepal (Hervieux et al., 2016). In a recent study, considering the elements as representative of cells with distinct cell types, FEM has been used to investigate the restoration of a regenerating root tip (Mathew et al., 2025). In FEM, plant tissues are treated as continuous elastic materials divided into small computational elements, enabling simulation of how forces and wall properties shape growth and deformation. Key stiffness parameters and wall thickness determine stress and strain patterns, but are difficult to measure precisely in living tissues. Even minor variations in these values can strongly affect model outcomes, highlighting the importance of accurate experimental inputs. Thus, while FEM offers a powerful tool to link mechanical forces with plant morphogenesis, its reliability depends on well-constrained parameters and appropriate simplifications of complex biological materials. Plant tissue is often multi-layered, with distinct viscoelastic properties of the cells belonging to respective tissue layers. Considering the differential viscosity in cell layers, the Vertex Element Model (VEM, **Table 1**- Eqn. 7), a VM extension, has been proposed (**Figure 2c**). In this approach, cells (which are represented as polygons in VM) are further divided into finite elements (e.g., triangles or rectangles), tracking both vertex positions and element deformations to model intra-cellular stress ( σ) and strain ( ϵ). This allows detailed mechanical analysis within individual cells and heterogeneous properties. While VM is computationally simple, VEM offers higher-resolution insights at greater computational cost. This model can simulate growth on realistic geometries using moderate computational resources. Using this model, it has been shown that reorientation of cellulose microfibrils, which can alter the viscoelastic properties of the cell wall, can regulate cell expansion across different tissue layers (Fozard et al., 2013).

For investigating the impact of cell wall elasticity and mechanical stress distribution during the tissue growth processes, a widely used modelling framework is the Mass-Spring Model (MSM, **Table 1**- Eqn. 8). This model represents a system of nodes, each with a position ( $x_{i}$), connected by springs, which approximates the cell wall with different mechanical properties, i.e., elastic or viscoelastic, to accurately model the mechanical response of the cell wall (**Figure 2d**). The net spring force on each node arises from its connections to neighbouring nodes, while viscous drag ( γ) opposes node motion, providing damping. The motion of each node is determined by the balance of spring, drag, and external forces. A significant application of this model is simulating the formation of leaves and phyllotaxis (de Boer et al., 1992). Further, a bi-layered version of MSM has successfully been applied to animate the dynamics of leaf movement, like the curling and wilting of leaves (Weise and Tusscher, 2019). In this model, the response of the system to internal pressure or external stress depends on spring stiffness, damping coefficient, and rest length. However, these parameters are often empirical and difficult to relate directly to measurable cell wall properties. Small changes in parameter values can lead to unrealistic oscillations or distortions, limiting quantitative accuracy. Therefore, while MSMs are useful for exploring qualitative behaviours and mechanical feedback in plant tissues, their predictive power is constrained by simplified assumptions and weak links to experimentally measurable biophysical properties. Apart from the formation of robust organs, plants show a remarkable pattern in terms of the positioning of aerial organs, such as the leaf or branching morphology of shoots or roots. In a pioneering modelling approach, the intricate branching morphology in plants has been modelled using L-systems (LSM), a rule-based mathematical formalism that simulates plant growth by encoding recursive branching rules (Boudon et al., 2012). Further, by incorporating genetic and environmental factors, the LSM has been utilized to explain how plants achieve diverse architectural forms in response to various developmental cues. An advanced implementation of the LSM allows the simulation of plant growth on curved, non-Euclidean surfaces using Riemannian geometry. This approach allows more realistic studies on morphogenetic patterning in plants, which involves nontrivial tissue curvature (Godin and Boudon, 2025). By changing the values of branching angle, internode length, and growth rate control overall shape, this model allows researchers to explore how local rules generate complex architectures. These parameters can be informed by experimental measurements, but natural variability across species, developmental stages, or environmental conditions can strongly influence model predictions. For example, a small change in branching angles or growth rates can dramatically alter the simulated plant form. Thus, while L-systems are powerful for visualizing and predicting plant architecture, their predictive accuracy depends on careful parameterization and may not capture mechanical feedback or tissue-level stresses.

When talking about patterns, a striking phenomenon unique to plants is phyllotaxis, which is the regular arrangement of lateral organs around a central axis. While active transport of auxin is known to regulate this phenomenon, a mechanistic understanding of the process has emerged only after a KM-based modelling study (Smith et al., 2006). This model is capable of generating different kinds of phyllotactic patterns (e.g., distichous, decussate, and tricussate patterns) based on auxin and PIN dynamics. Interestingly, this model can reproduce experimentally measured divergence angle, i.e., the angular separation between successive primordia as they emerge from the shoot apex. In plants, the function of organs is strongly linked to their structures, i.e., leaves have flat surfaces to get maximum exposure to sunlight, or roots have pointed shapes to penetrate through the soil. To study the relationship between the function of organs and their structures, a more complex Functional-Structural Plant Model (FSPM) has been introduced, which uses LSM as a foundation (Godin and Sinoquet, 2005). This model has gained prominence due to its ability to simulate organ-level growth dynamics and whole-plant responses to environmental factors, e.g., light or water. Notably, FSPM has also been used to investigate plant organogenesis (Vos et al., 2010). So far, we have talked about how cell-cell interaction drives organ morphology and how this is related to organ function. However, the aerial organs also interact with the environment for light and nutrient absorption. A sophisticated way to model such multiscale interactions is to use Agent-Based Models (ABM), which can simulate cell-cell interactions at the tissue level and plant-plant and plant-environment interactions at the population and ecology level (Zhang and DeAngelis, 2020). In recent work, LSM has been combined with ABM for versatile representations of the environment and root growth dynamics (Mußmann et al., 2024). In summary, the models investigated in this section demonstrate how coupling between geometry, biomechanics, and genetic instructions interacts to direct controlled growth and patterning that drives plant morphogenesis. The modelling frameworks also illustrate an emerging cycle of model-based prediction and experimental validation, thus progressively bridging computational predictions with real biological systems.

## Metabolism and resilience

**Figure 3:**
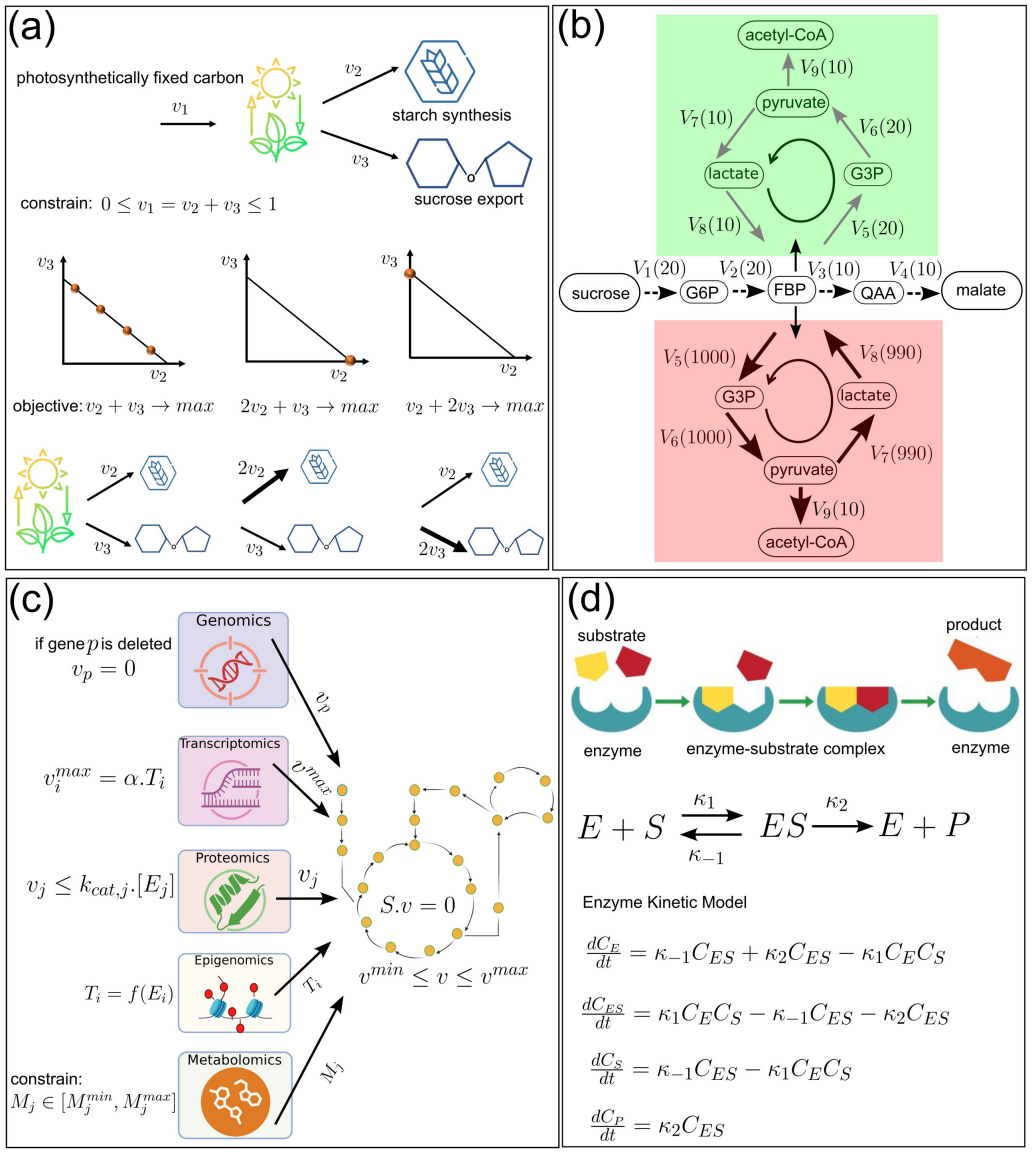
Models of metabolic fluxes and enzymatic kinetics. **(a)** Constraint-based optimization model for flux distribution in plant metabolism. A carbon flux space defined by two pathways (v_1_ and v_2_) ) with constrain (0 ≤ v1 + v2 ≤ 1) (1) linear objective v_1_ + v_2_ → *max* yields multiple optima (orange dots), (2) weighted objective 2v_1_ + v_2_ → *max*, resulting in a single optimum, favouring flux through v_1_, and (3) weighted objective v_1_ + 2v_2_ → *max*, favoring flux through v_2_. **(b)** Flux balance analysis model for understanding carbon (e.g., sucrose) allocation. Under stressed conditions (shown in red colour), carbon is routed through a nonlinear flux cycle (*V*_5_-*V*_8_, flux ~ 1000), incurring suppression of acetyl-CoA flux (*V*_9_), resulting in low biomass production. In the absence of stress (shown in green colour), routing follows a linear flux cycle (*V*_5_-*V*_8_, flux ~ 10), causing increased acetylCoA flux (*V*_9_), thus resulting in enhanced biomass production. **(c)** Genome-scale metabolic modelling with a core metabolic network interfaced by omics layers. Genomic module constrains fluxes with gene deletions disabling associated reactions (v_p_ = 0). Transcriptomic module puts upper limit on fluxes (v_i_^max^). The proteomic module refines flux predictions (v_j_) by relating enzyme (*E*_j_) abundance and turnover rates to catalytic capacity (K*_cat,j_*). Epigenomic modules influence flux via regulation of gene accessibility and expression (T_i_), while metabolomic modules guide objective function formulation (M*_j_*). Together, they feed into the core FBA formulation, defined by mass balance at steady state (S. v =0), and bounded by physiological constraints (v_min_ ≤ v ≤ v_max_). **(d)** Enzyme-kinetics model, where an enzyme (E) binds to a substrate (S) to form a transient enzyme–substrate complex (ES), which subsequently dissociates to product (P) and the free form of the enzyme. Dynamics of the concentration S, E, ES, and P can be described through Michaelis–Menten kinetics, e.g., ODEs-based kinetic model discussed in **Figure 1a**, with reaction rate depending on various kinetic rate constants (K_1_, K−_1_, and K_2_).

Plant growth leads to an increase in cell number and cell volume, thus posing a challenge in managing limited resources to power the associated metabolic processes. In this section, we delve into the modelling studies that help in understanding the strategy through which plants balance limited metabolic resources while maintaining growth and stress resilience. We have discussed how mechanical forces, i.e., turgor pressure and cell wall stress, are key in shaping plant growth and form. These forces are regulated by various hormones, osmolytes, and structural proteins and are produced or modulated by various metabolic pathways, establishing a dynamic feedback loop between metabolism and cellular mechanics. Interestingly, in spite of the limited availability of metabolic resources, cellular functions remain feasible. To understand the underlying mechanism, the Constraint-Based Models (CBMs) have been introduced, where the flux vector. v is constrained by the stoichiometric balances s.v=0  and flux bounds $v_{min}\leq v\leq v_{max}$ with s  being the stoichiometric matrix (**Table 1**- Eqn. 9). These biophysical and metabolic constraints ensure minimal cell functionality in the absence of sufficient metabolic resources (**Figure 3a**). Using this model, it has been shown that metabolic networks in certain plants have natural advantages over others, allowing them to thrive under challenging conditions by reducing photosynthesis losses and improving the efficiency of carbon assimilation (Wang et al., 2012). CBM models use Flux Balance Analysis (FBA) to predict metabolic flux based on the stoichiometric metabolic model (**Figure 3b**), and help to understand how plants allocate nutrients, enzymes, and energy for optimal growth and adaptation (Yuan et al., 2016). The structural composition of the cell wall determines the load-bearing capability of a plant, e.g., plants with wood-bearing cells are more resilient against strong winds. In this context, an important application of FBA can be found in understanding monolignol biosynthesis, a crucial process that determines the structural composition of plant cell walls (Wang et al., 2019). In this work, dynamic changes in metabolic flux within wood-forming cells have been captured by integrating experimental data into the model. Another important application of FBA can be found in identifying the role of inorganic phosphate metabolism in sustaining seed metabolism under limited oxygen demand (Grafahrend-Belau et al., 2009). The predictive power of FBA critically depends on the choice of objective function and on how well the model reflects biochemical constraints. Objective functions such as biomass maximization or minimal flux assumptions may lead to different flux distributions. For instance, changing biomass composition or objective criteria yields appreciable shifts in flux predictions, particularly in glycolysis and the Calvin–Benson cycle (Yuan et al., 2016). Moreover, approaches that account for enzyme cost or proteome allocation further refine flux predictions. For instance, an FBA variant has been proposed that incorporates enzyme cost weighting, effectively penalizing flux states that require high enzyme investment (Cheung et al., 2015). The FBA model optimizes metabolic flux distribution under fixed conditions; however, fluctuations in environmental conditions introduce dynamic shifts in metabolic processes. To capture these dynamic metabolic shifts, an FBA model has been coupled with a dynamic photosynthesis model (Shameer et al., 2022). This hybrid modelling approach has enabled the simulation of dynamic carbon assimilation, starch accumulation, and associated electron flux. Notably, in recent developments, such hybrid modelling concepts have been further extended, e.g., a kinetic photosynthetic modules (i.e., ePhotosynthesis) has been integrated into a crop growth model, and a thorough analysis identified key enzymes (e.g., PGK, PRK) whose perturbation increases assimilation by 8% and yield by 6.7% (He et al., 2024). Building on such developments, comparative analyses of objective functions in plant-specific metabolic models have revealed that the optimization criterion chosen, whether biomass accumulation, energy efficiency, or minimal nutrient consumption, profoundly influences predicted flux distributions and physiological interpretations (**Table 2**). FBA or its parent CBMs primarily account for metabolic flux constraints, but neglect enzyme capacity constraints as they do not explicitly consider the proteome allocation required to sustain these fluxes.

**Table 2:** Objective functions and their metabolic trends.

Objective function	Metabolic trend	Approximate outcome	Interpretation
Biomass Maximization (Yuan et al., 2016)	Flux through glycolysis & Calvin cycle ↑	Substantial increase in anabolic fluxes	Growth-favorable metabolic state
ATP Yield Maximization (Amthor, 2023)	Oxidative phosphorylation & TCA flux ↑	Energy-efficient flux allocation	Prioritizing ATP production over growth
Minimal Nutrient Uptake (Baghalian et al., 2014)	Secondary metabolism ↑, growth flux ↓	Reduced biosynthetic flux, more stress metabolism	Adaptation under nutrient limitation
Enzyme/Proteome-Constrained FBA (Cheung et al., 2015)	Redistribution under a limited enzyme budget	Noticeable flux rerouting when enzyme costs are penalized	Trade-off between growth and enzyme usage constraints

Summary of representative modelling outcomes illustrating how different objective functions shape flux allocation, energy balance, and stress-response behaviours in plants.

To address this, Proteome Constrained Models (PCM, **Table 1**- Eqn. 10), an extension of CBM, have been introduced. This extension forces flux trade-offs and more realistic predictions under limited protein resources (Chen and Nielsen, 2021) and enables capturing the trade-offs between metabolic flux capacity and proteome resource expenditure, rendering more biologically realistic predictions of cellular metabolism under various environmental and physiological conditions. While CBMs incorporate constraints on metabolic fluxes, the PCMs impose constraints on protein synthesis and resource allocation, ensuring that metabolic predictions align with cellular proteomic capacity. In an important application of this model, proteomic information from different parts of the plant has been integrated, enabling whole-scale modelling of plant growth and development (Gao and Zhao, 2024). The model predicted that under nutrient-limiting conditions, proteome constraints force the plant to reprioritize metabolic pathways, favouring essential maintenance and stress response functions over growth-related processes. This leads to metabolic flux rerouting consistent with experimental observations of metabolic adaptation during stress. Essentially, PCMs enhance our understanding of resource-limited cellular processes by refining metabolic predictions through protein allocation constraints. However, PCMs are still limited to specific cellular processes and do not comprehensively capture the full metabolic potential of an organism.

The limitations of PCMs have been overcome by Genome-Scale Metabolic Models (GSMMs), which capture large-scale metabolic networks by integrating genomic, transcriptomic, proteomic, and metabolomic data to simulate system-wide biochemical reactions (**Figure 3c**). By mapping the molecular-scale biochemical reactions occurring within cells, GSMM offers a comprehensive approach to understanding the metabolism of plants in a more holistic fashion. By integrating genome annotation, biochemical pathways, and stoichiometric constraints, GSMMs enable the quantitative prediction of metabolic fluxes under diverse environmental and genetic conditions. These models have been instrumental in exploring how plants reprogram metabolism during growth, development, and responses to abiotic stresses such as drought, salinity, and nutrient limitation (Cloutier et al., 2021). For instance, recent plant-specific GSMMs have been used to simulate carbon and nitrogen fluxes, identify key enzymes governing biomass accumulation, and elucidate metabolic trade-offs under light and nutrient stress (de Oliveira Dal’Molin and Nielsen, 2013). Moreover, the integration of GSMMs with transcriptomics, proteomics, and metabolomics data allows for the reconstruction of condition-specific metabolic states, providing insights into the coordination between metabolism and gene regulation during developmental transitions and pathogen defence (Mangaravite et al., 2025). These advances position GSMMs as essential frameworks for predictive biology, metabolic engineering, and sustainable crop design. In another notable application of GSMM-based simulation of biomass production, it has been shown that only a small fraction of the metabolic reactions were essential, underscoring the network’s efficiency (Poolman et al., 2009). Further, GSMMs have been utilized to maximize the biomass production of key metabolites and improve stress tolerance in plants (Cunha et al., 2023). GSMM and CBM assume a steady state condition, i.e., the concentration of metabolites does not change over time. Therefore, these models fail to capture transient metabolic responses over time.

To overcome these limitations, an alternative modelling approach is to use the Kinetic Model (KM), which incorporates dynamic reaction kinetics and allows time-dependent simulations in metabolic concentration (Rohwer, 2012). KMs provide detailed insights into network control and responses to genetic perturbations, allowing estimations of metabolic flux at varying intermediate and enzyme concentrations (Colón et al., 2010). Moreover, KMs provide critical insights into carbon flux regulation, essential for optimizing biofuel production (Nag et al., 2011). In a metabolic process, the efficiency of biochemical reactions is determined by metabolic enzymes, whose availability changes dynamically. To understand the role of metabolic enzymes in the efficiency of metabolic reactions, Enzyme Kinetic models (EKMs, **Table 1**- Eqn. 2) have been introduced (**Figure 3d**). EKMs offer a more intricate representation of enzyme-mediated reactions; however, they involve additional kinetic parameters whose estimation requires extensive experimental data. In fact, EKMs are extensively utilized to identify potential targets for improving yields in agricultural fields to modulate the biomass production rate (Hu et al., 2022). It is well known that the enzyme activity is controlled by gene regulatory networks (Keurentjes et al., 2008), which can be modelled by BNMs. A recent work highlights the transcriptional regulation and activity modulation of key photosynthetic enzymes in plants, focusing on the integration between genetic regulation and environmental response (Steichen et al., 2024). The study designed a light response system to monitor and analyse the transcriptional changes of photosynthetic enzymes and regulatory proteins in response to high and low light conditions, tracking carbon assimilation and metabolic shifts. Further, BNMs have important applications in synthetic biology in designing genetic circuits, which utilize bacterial allosteric transcription factors to regulate gene expression based on ligand availability, allowing precise control of metabolic pathways (Ferreira and Antunes, 2024). In summary, the models revisited in this section capture both the efficiency and flexibility of plant biochemical networks, revealing how plants dynamically adjust carbon fixation, proteome allocation, and metabolic fluxes to optimize growth and show resilience by withstanding fluctuations in resource availability.

## Physiology and functional sustainability

**Figure 4:**
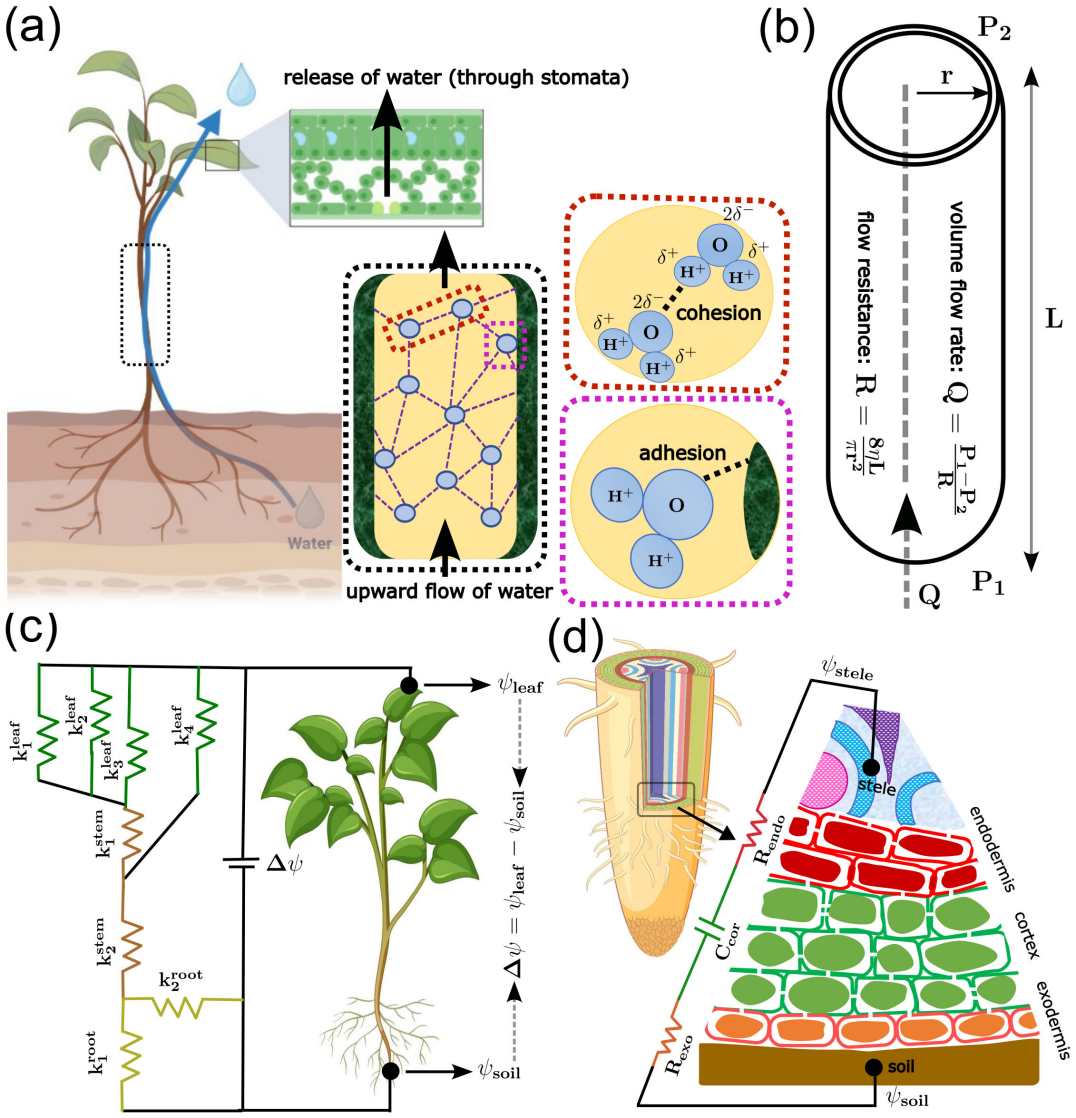
Models of the physiology of water and nutrient uptake. **(a)** Cohesion–Tension theory of water movement against gravity. Continuity of the water column in the xylem (highlighted in black dashed box) is maintained by cohesion force, which is the mutual attraction between water molecules (highlighted in the red dashed box), and adhesion force, which is the interaction force between water molecules and hydrophilic cell walls (highlighted in the magenta dashed box). **(b)** Hagen–Poiseuille model of water transport. In the cylindrical conduit (analogous to xylem vessels), volumetric flow rate (Q) is a function of the pressure gradient (P_1_ − P_2_) and flow resistance (R), which depends on the dynamic viscosity of xylem sap (h), along with the length (L) and radius (r) of the conduit. **(c)** Ohm’s law-based model for water transport. Water flow is governed by the difference in water flux between the soil and the leaf, Δψ = ψ_soil_ − ψ_leaf_, which is modulated by the hydraulic conductance (inverse of resistance) of the root (k_i root_), stem (k_i stem_), and leaf (k_i leaf_), capturing the plant hydraulic architecture and pathway. **(d)** Hydraulic-electrical circuit-based model for water movement from soil to the stele (xylem). Similar to panel (c), a difference in water potential between the medium (ψ_m_) and the stele (ψ_stele_) drives the water transport. The resistance to the flow comes from the intermediate exodermis (R_exo_) and endodermis (R_endo_) tissue layers. Additionally, transient storage of water by the cortex layer is modelled by capacitance (C_cor_).

Plant survival and efficient adaptation to environmental changes depend not only on metabolic efficiency but also on the physiological processes that drive sustained transport of metabolic resources, e.g., transporting water and nutrients across different body parts. In this section, we turn to models that explain critical physiological processes. In the context of water transport, one of the pioneer modelling approaches is the Hagen-Poiseuille equation (**Table 1**- Eqn. 11), which describes how a fluid flows steadily through cylindrical tubes (**Figure 4a**) and enables estimation of the volumetric flow rate Q based on the pressure difference between the two ends of the tube ( ΔP), tube radius ( R), tube length ( L), and fluid viscosity ( η). This equation has been utilized to study xylem structure in balancing hydraulic efficiency under water stress conditions (Tulik and Marciszewska, 2013). This formulation emphasizes the principle of hydraulic capacity, as the conductivity of xylem vessels depends on lumen size and fluid properties. For instance, it has been demonstrated that variation in ion concentrations alters the viscosity of the fluid, influencing hydraulic conductivity and, therefore, the effective resistance of the transport pathway (van Ieperen et al., 2000). This work highlighted that ion-mediated changes in xylem flow dynamics play a crucial role in plant water transport, particularly under environmental stress. However, this model assumes a continuous, pressure-driven flow of liquid, which does not fully capture long-distance water transport, which requires a continuous flow of water against gravity. This limitation can be addressed using the Cohesion-Tension Theory (C-T theory) based hydraulic model (**Figure 4b**), which reveals that the upward flow of water against gravity is due to cohesion between water molecules and their adhesion to the xylem wall (Tyree, 1997). In an interesting application of the C-T theory, modifying water adhesion via altering the xylem sap properties predicted associated changes in hydraulic conductivity and flow stability, establishing the safety-efficiency trade-off in water transport (Schenk et al., 2017). To maintain optimal water transport, plants must manage their water usage. To analyse how plants regulate water loss, by considering water transport as an electrical analogue, an Ohm’s law-based hydraulic model has been developed (**Figure 4c**). In the soil-plant-atmosphere continuum (SPAC), the interconnected pathway through which water moves from soil, through plants, and into the atmosphere via transpiration, this model combines hydraulic resistance, stomatal response, and environmental effects (Manzoni et al., 2013). Root water uptake significantly varies under changes in environmental factors. To explain the relation, the Composite Transport Model has been introduced (Steudle, 2000). This model provides insights into how plants adapt to drought and stress conditions by adjusting water uptake efficiency, and by extension, can predict vulnerability curves, which describe how embolism and cavitation reduce xylem function under stress. Together, these models provide a mechanistic understanding of efficient water transport and optimal water usage under different environmental conditions.

**Figure 5:**
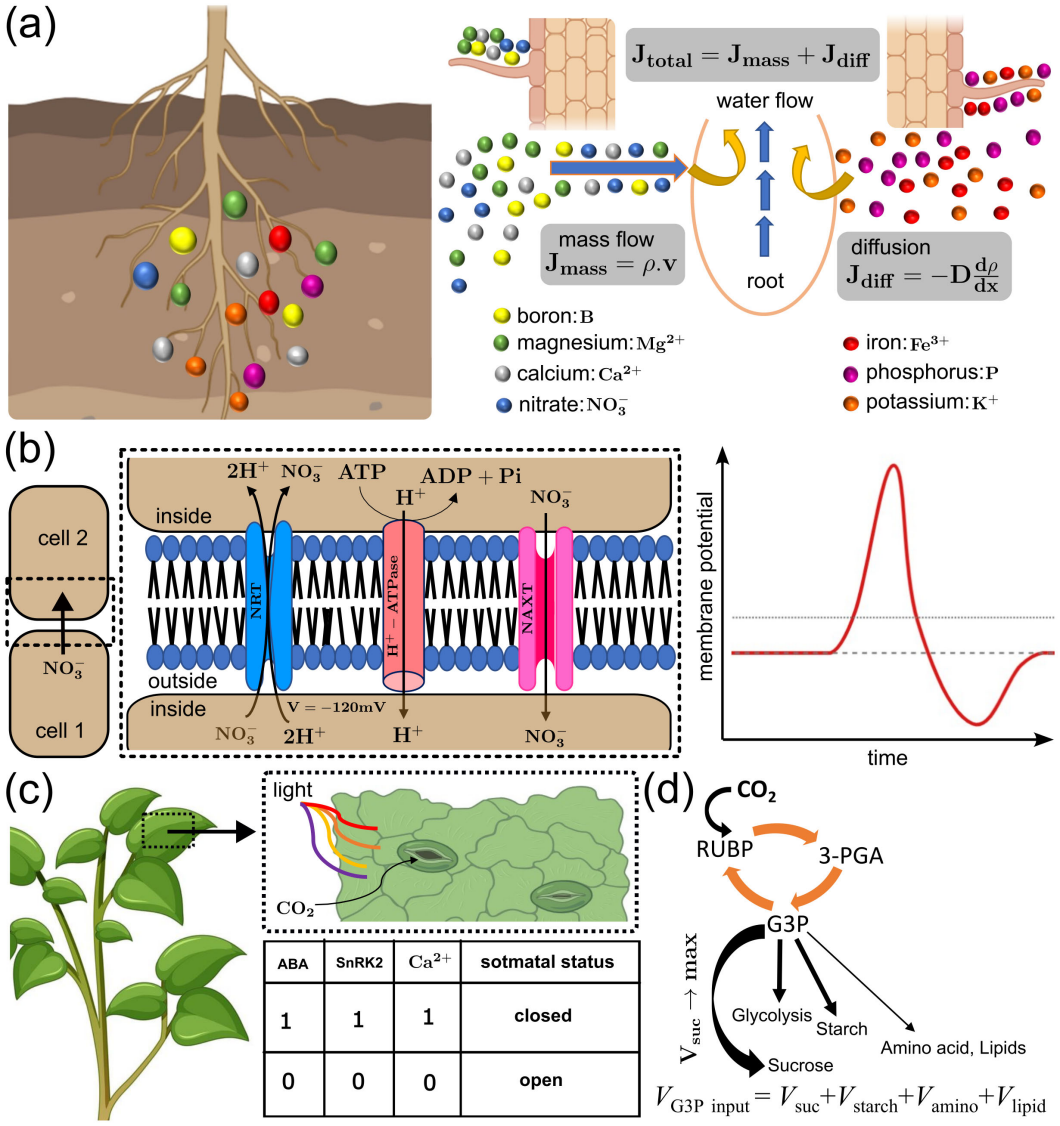
Models for ion transport, stomatal regulation, and metabolic carbon flux. **(a)** Dynamic Nutrient Uptake Model for nutrient transport. The long-range transportation of mobile nutrients (e.g., NO_3-_, Ca²^+^, etc.) happens through mass flow with flux J_mass_, governed by the concentration (ρ) of nutrients and the velocity (v) of water from the soil to the root surface. The short-range movement of immobile nutrients (e.g., P, K^+^, etc.) happens through diffusion with flux J_diff_ , governed by the diffusion coefficient (D) of soil and the concentration gradient (*d*ρ/*dx*) of nutrients in the soil. Total nutrient flux (J_tot_) is simply the addition of J_mass_ and J_diff_. **(b)** Hodgkin–Huxley-type model for the voltage-gated transport of nitrate (NO_3-_) across membranes (highlighted in black dashed box). Multiple ion channels mediate NO_3-_ uptake and redistribution. Membrane potential vs. time plot (red curve on right), showing a stable, highly negative membrane potential before NO_3-_ addition. Upon entry of NO_3-_ via H^+^/NO_3-_ symporters, the influx of anions causes a rapid depolarization, making the membrane potential less negative (dense thin dashed line indicates zero reference). Over time, the H^+^ATPase and other ion channels restore the negative potential, producing a recovery phase toward the original membrane potential (sparse thick dashed line). **(c)** Boolean network model for understanding the regulation of stomatal aperture, a door for accessing atmospheric CO2, which is a prerequisite for photosynthetic carbon assimilation. The black dashed box highlights the entry of atmospheric CO2 into the leaf through open stomata, and the truth table (with ABA, SnRK_2,_ and Ca²^+^ as nodes) explains the Boolean rules for the closing/opening of stomata. **(d)** Stoichiometric framework for the conservation of carbon flux during photosynthetic carbon assimilation. The CO2 accessed from the atmosphere is assimilated into the Calvin cycle (orange arrows), where it is fixed into 3-PGA, converted into G3P, and distributed among competing biosynthetic pathways for production of sucrose, starch, amino acids, and lipids. The optimization objective is denoted as Maximize V_suc_, directing flux preferentially toward sucrose synthesis.

While the transport of nutrients can be associated with the transport of water, the mechanism by which the intake of nutrients happens via the root epidermal surface is complex. To account for this complexity, a Hydraulic-electrical circuit-based model (**Table 1**- Eqn. 12) has been developed (**Figure 4d**), in which water flow is treated like electric current, where volumetric flow rate Q is analogous to current, water potential difference ( Δψ) to voltage, hydraulic resistance ( R) to electrical resistance, and capacitance ( C) to water storage capacity. This model simulates the absorption of nutrients from the soil by different crops at various stages of growth (Oates and Barber, 1987). An extended version of this model accounts for the dynamic growth of roots and the associated competition in nutrient uptake (Reginato et al., 2000). To account for changes in nutrient levels in the soil and other environmental conditions, a Dynamic Nutrient Uptake Model (**Table 1**- Eqn. 13) has been proposed (**Figure 5a**), in which the nutrient uptake assumes that the transport of nutrients in soil occurs only through the continuous soil pore space via flux of diffusion ( $J_{diff}$) and mass flow ( $J_{mass}$) (Leitner et al., 2010), and total nutrient flux is given by $J_{total}=\;J_{diff}+J_{mass}$, describing overall nutrient uptake dynamics. The mass flow dominates when water movement is high, carrying dissolved nutrients (parameterized by its density, ρ) toward the root surface. On the other hand, diffusion (parameterized by diffusion coefficient, D) becomes significant when nutrient gradients exist around the root zone, especially for less mobile ions. To simulate the enhanced nutrient acquisition capability, a hybrid reaction-diffusion model has been developed that integrates root architecture, exudation rates, and soil nutrient diffusion coefficients. This model showed how nutrient-enriched zones are formed, optimizing resource allocation in nutrient-deficient environments (Zygalakis and Roose, 2012). The general source of the nutrients is the soil particles, and the rate of nutrient release from soil particles depends on diffusion. Notably, nutrients can move through diffusion both within and between soil particles. Considering this aspect, a dual porosity model has been introduced to understand the role of root hairs in nutrient uptake (Zygalakis et al., 2011), which revealed that root hairs significantly enhance nutrient uptake, especially due to slow nutrient release from soil particles. This model also predicted that longer root hairs have a greater impact on uptake efficiency than increased hair density, particularly in dry soils where diffusion is more restricted. Moving forward, a multiscale analysis of nutrient uptake examined different scaling relationships between root hair radius and inter-hair distance (King et al., 2021). The study highlights the role of root hair spacing in nutrient acquisition and provides insights for optimizing root architecture to enhance nutrient absorption efficiency. So far, we have discussed one way of nutrient uptake, i.e., from soil to the plant tissue. However, there can be an exchange of nutrients between the plant and other organisms, e.g., fungi. Considering this, a computational framework has been developed to investigate nutrient exchange dynamics, where plants and fungi trade sugars and phosphate through proton-coupled transporters that operate at the periarbuscular (Schott et al., 2016). The model suggests that apparent cooperation arises from competitive interactions for shared resources rather than pure mutualism. A complementary thermodynamic model explores nutrient exchange across the two-membrane system, identifying proton-driven transport as central for efficient phosphate and carbon transfer (Dreyer et al., 2019). Together, these models provide mechanistic and energetic insights into plant-fungus nutrient exchange. While uptake is important, the cells must maintain ion and nutrient homeostasis, and recent modelling studies have shown that this process can be coordinated by a network of transporters, known as homeostats. This work reveals that instead of single types, multiple energetically distinct transporters are essential for potassium ion (K^+^) homeostasis and stable nutrient regulation (Dreyer, 2021; Dreyer et al., 2024). An extension of this framework explored the dynamic responses of K^+^ homeostats to electrical and chemical stimuli, revealing fast and slow regulatory phases (Contador-Álvarez et al., 2025). These studies present a blueprint for understanding how combinations of transporters work together, rather than relying on single proteins, to support plant growth, nutrient uptake, and stress responses.

Notably, active transport of nutrients between neighbouring cells happens through the membrane potential, which is created and maintained by a voltage-gated mechanism that regulates the opening and closing of associated ion channels. This movement of ions across membranes through specialized transporters and channels can be explained by electrophysiological models. For example, the Hodgkin-Huxley model (**Table 1**- Eqn. 14), originally developed for neurons (**Figure 5b**), has been adapted to describe ion flux dynamics in plant cells, particularly how voltage changes influence ion channel activity (Sukhova et al., 2017). This model explains how the membrane potential ( $V_{m}$​) of a plant cell changes due to ion flux across the plasma membrane. The membrane acts as a capacitor ( $C_{m}$​) that stores electrical charge, while ion channels behave as conductance that control ion flow. The leak conductance (​ $g_{L}$) and its reversal potential (​ $E_{L}$) represent passive ion movements that stabilize $V_{m}$. The parameters $g_{L}$​, $C_{m}$​, and $E_{L}$ ​ critically shape membrane behaviour by determining how fast and strongly voltage changes occur. A higher $g_{L}$ ​ or altered $E_{L}$ shifts the resting potential, while an increased $C_{m}$ ​ dampens rapid voltage fluctuations, influencing ion channel activation dynamics. In this regard, the Zhao model provides a deeper insight into how membrane potential and electrical signals regulate nutrient uptake and redistribution (Zhang et al., 2021). Further modelling suggests that these electrical events may be accompanied by ion waves that modulate excitability across tissues (Cuin et al., 2018). Further, combining experiment and computational modeling work reveals that in plant vascular tissues, K^+^ ions circulate in the phloem and act like a potassium battery, storing energy that can be used locally to overcome energy shortages during nutrient transport (Gajdanowicz et al., 2011). Through nutrient uptake, plants receive key elements to sustain and optimize photosynthesis, an essential physiological process for their survival and reproduction. In plants, the rate of photosynthesis determines the efficiency of nutrient-to-food conversion, in which light energy is converted to chemical energy. The rate of photosynthesis can be described by the Farquhar-von Caemmerer-Berry model (Farquhar et al., 1980), which offers insights into how plants optimize carbon fixation under different environmental conditions. This model has been used to investigate the role of light intensity on photosynthesis, e.g., a sudden decrease in the net photosynthesis rate versus light intensity, also known as the Kok effect (Yin et al., 2020). It is well documented that CO_2_ is essential for the Calvin cycle in photosynthesis, and abscisic acid-driven stomatal closure is known to limit this CO_2_ uptake by reducing the stomatal aperture. In this regard, a Boolean network-based computational tool (**Figure 5c**), Boolink, has been developed to model abscisic acid-driven stomatal closure (Karanam et al., 2021). Another important computation tool that allows mechanistic modelling of guard cells is the OnGuard platform, which integrates ion transport, signalling, and osmolyte metabolism (Hills et al., 2012). It reproduces realistic stomatal opening and closing through detailed biophysical simulations that conserve charge and mass. The newer OnGuard3e extends this framework to whole-plant scales, linking guard cell dynamics with photosynthesis and water-use efficiency. These tools provide a powerful bridge between molecular mechanisms and plant-environment interactions (Nguyen et al., 2023). Stomata regulate the balance between CO₂ uptake for photosynthesis and water loss through transpiration. A widely used formulation that relates stomatal conductance to photosynthetic rate, atmospheric CO₂ concentration, and relative humidity is the Ball-Berry model (Ball et al., 1987). Coupling this model with the Farquhar photosynthesis model enables linking environmental drivers to leaf-level gas exchange, enabling predictions of assimilation, transpiration, and water-use efficiency under varying conditions. Importantly, photosynthesis drives metabolic fluxes by supplying fixed carbon and energy that fuel and regulate metabolic pathways. Considering this, a metabolic flux analysis has been employed to demonstrate how adjustments in photosynthetic rates affect carbon fluxes, which play a crucial role in biomass (sucrose) production (**Figure 5d**), thus providing insights into optimizing plant growth under changing environmental conditions (Xu et al., 2025). However, quantitative analyses in photosynthesis models show that modest changes in enzyme capacity can produce substantial changes in flux. For example, resource-allocation analyses identify Rubisco and other Calvin-cycle enzymes as major controllers of CO₂ assimilation (Zhu et al., 2007). Guard-cell kinetic frameworks likewise demonstrate that ion-pump parameters act as fragile nodes whose modest perturbation disproportionately alters ion fluxes and stomatal behaviour (Hills et al., 2012).

Functioning of the various plant organs requires energy, which is typically allocated through the distribution of sucrose from the source (leaves) to the sink (organs) by phloem tissue. Modelling of this distribution process has long been centred on the Münch pressure-flow hypothesis, i.e., the hydrostatic pressure that drives nutrient and water flow between the source and the sink is generated by osmolytes such as sucrose (Münch, 1930). For example, the origin of the driving force behind the bulk flow through sieve tubes can be explained by osmotically driven pressure differences between loading and unloading sites. More recent models couple this with xylem water transport and explicitly include loading/unloading kinetics and sieve tube anatomy to predict sugar fluxes, pressure profiles, and their interaction with transpiration (Knoblauch et al., 2016). Such models underscore the principle of segmentation in transport networks, where source and sink regions function as discrete but interconnected modules. Taken together, the modelling frameworks discussed in this section highlight how plants optimize hydraulic capacity, regulate resistance, employ osmotically driven bulk flow, and achieve functional segmentation to maintain resilience under diverse environmental conditions. Integrating multiple modelling approaches within the broader SPAC perspective, from nutrient uptake and transport to electrophysiology and photosynthesis, researchers can develop robust strategies for improving plant resilience and productivity.

## Next-generation applications in biotechnology

Progress in plant science and engineering has revolutionized our ability to manipulate plants for agricultural productivity. By integrating different modelling approaches, researchers can now design targeted interventions to increase crop yield, reduce pests, and develop innovative biotechnological applications for crop breeding. In this section, we aim to elucidate the potential use of existing biophysical models in cutting-edge bioengineering applications. Crops suffer yield loss under drought and nutrient stress. Here, a synthetic genetic circuit, a form of BNMs, can help find strategies to optimize water and nutrient use in real time. Based on this concept, we can develop a functional modelling approach that couples the C-T theory for water transport with BNMs controlling both root uptake and stomatal conductance. This will enable the design of crops with programmable water and nutrient management. Crop yield can also be compromised by pests, e.g. presence of *Helicoverpa armigera*, the cotton bollworm, which reduces cotton yield. To mitigate this, biotechnologists have developed transgenic cotton plants that express toxin genes derived from *Bacillus thuringiensis* (Bt). In this technology, the Bt-toxin genes are inserted directly into the cotton genome, enabling the plant to produce the toxin that is lethal to *Helicoverpa armigera*, thereby protecting the crop from compromising growth and yield. This gene manipulation technology can be advanced by combining BNMs and GSMMs, where the former will allow simulation of gene regulatory networks to predict the expression dynamics of the Bt-toxin genes within the plant, and the latter can analyse metabolic interactions within the plant metabolism itself to optimize energy and resource allocation for toxin production. Together, this integrated modelling approach can enhance the design of transgenic varieties that maximize pest resistance without the need for external pesticide application. Biophysical models can also be applied to engineer genetically modified crops to maximize biomass production under varying environmental conditions. Here, we can develop a cutting-edge computational framework to provide strategic guidance for engineering genetically modified crops, where the model will integrate FBA, PCMs, and BNMs. In this model, FBA will help optimize metabolic fluxes under resource constraints, while PCMs will account for protein synthesis and resource allocation. On the other hand, BNMs will help simulate gene regulatory networks to control gene expression and relevant environmental responses. This hybrid modelling approach contains the necessary ingredients for designing genetically modified crops optimized for maximum biomass production.

Drawing on the latest breakthroughs in plant computational biology, artificial intelligence (AI), and machine learning (ML), are increasingly reshaping modelling frameworks by tackling persistent challenges in multi-scale integration, parameter optimization, and predictive analytics. Deep learning models based on AI now enable the extraction of high-throughput phenotypic traits from intricate image data sets, thus enhancing genotype-phenotype mapping as well as crop trait prediction in precision agriculture (Murphy et al., 2024). ML also allows automatic parameter estimation and sensitivity analysis in large-scale biophysical and biochemical models, thus greatly enhancing calibration speed as well as predictive accuracy (Akhavizadegan et al., 2021). Recent advances also introduced hybrid architectures, including physics-informed neural networks (PINNs) and ML-augmented mechanistic models, that capture nonlinear dynamics and biofeedback that traditional equations struggle to reproduce (Ren et al., 2025). By integrating experimental data with biological priors, these hybrid models provide interpretable, scalable, and computationally efficient pipelines for simulating plant growth and stress responses across scales (Murmu et al., 2024). Collectively, these advances highlight AI and ML as complementary tools rather than replacements for traditional modeling frameworks, pointing toward a next generation of predictive, interpretable, and multi-scale plant systems biology.

## Discussion

In this review, we have highlighted how different modelling frameworks, ranging from cellular-scale gene regulatory models to tissue-scale mechanical models to whole-plant physiological models, offer complementary insights into plant growth and development. These models provide a systems-level understanding, thus linking molecular mechanisms with behaviour at the organismal level. For example, mechanical models, such as the VM and FEM, reveal how changes in local cell geometry and mechanical properties of the cell walls significantly influence outcomes of global morphogenetic events, e.g. organ morphology. In this context, the use of mechanical models reveals the importance of accounting for cell-cell coordination and physical constraints toward understanding the shaping of plant morphology. Further, coupling these mechanical models with KMs that describe auxin transport and BNMs that describe gene regulatory networks provides a coherent mechanistic explanation for cell differentiation, tissue patterning, and developmental robustness. To uncover strategies used by the plants to manage resource allocation under varying environmental conditions and diverse physiological constraints, metabolic models such as FBA, PCMs, and GSMMs have been developed. Many of these models are quantitative and play a crucial role in identifying metabolic trade-offs and bottlenecks in energy and nutrient use. Importantly, the application of these models spans from the prediction of biomass production to developing bioengineering strategies for stress tolerance and yield improvement. On the physiological aspect of plant growth and development, models of water and nutrient transport elucidate the principles governing long-distance resource movement and regulation. Notably, hydraulic models with their foundation on the C-T theory, along with nutrient uptake models, reveal how plants balance efficiency and safety when managing optimal nutrient resources. These models are no longer peripheral tools but central components of modern plant science. They not only enhance mechanistic understanding but also offer deeper insights for crop improvement, sustainable agriculture, and environmental resilience. Continued refinement and integration of these models will be key to translating plant biology into predictive and data-driven science.

While advances in modelling approaches have revolutionized our approach to studying plant growth and development, there are several limitations. For example, a lot of the models that we discussed operate under theoretical assumptions and often fail to capture the spectrum of variability observed in natural systems. Another challenge is the scalability of models across different biological levels, i.e., from intracellular processes to tissue dynamics to whole-plant response to environmental cues. Though several attempts are being made to develop multiscale modelling frameworks, a key challenge remains their validation against parameterization. Additionally, to be quantitative, many of these model frameworks need to be integrated with experimental data while maintaining optimal computational cost. Here, advances in imaging, single-cell omics, and machine learning are expected to accelerate this integration, thus enabling predictive modelling of complex traits. Further, synthetic biology approaches could offer the opportunity to implement and experimentally test model hypotheses, which has the potential to close the gap between model implementation and experimental observations.
